# Biocatalytic Radical C(sp^3^)–N Coupling via Active Site Templating

**DOI:** 10.21203/rs.3.rs-9204910/v1

**Published:** 2026-04-03

**Authors:** Zayed Alassad, Todd K. Hyster

**Affiliations:** 1 Princeton University, Princeton, NJ 08544, USA.

## Abstract

Stereoselective nucleophilic substitution to access α-tertiary amines relies on copper-catalyzed radical approaches in which the substitution is mediated by metal–anilide coordination.^1^ These systems, however, are constrained by competing arene radical alkylation pathways.^2^ Here we report a distinct photoenzymatic mechanism for enantioconvergent nucleophilic substitution that operates without metal coordination to the nucleophile. Six rounds of protein engineering yielded a variant of a flavin-dependent oxidoreductase that promotes C(sp^3^)–N coupling between tertiary alkyl halides and simple anilines in good yields, with high chemoselectivity for N- over C-alkylation and high enantioselectivity across a broad substrate range. Multivariate statistical analysis, density functional theory, and mechanistic experiments show that the active site templates π-stacking, hydrogen-bonding, and water-bridged interactions between a tertiary radical and the aniline lone pair to generate an intermolecular n→SOMO hyperconjugative complex that is energetically disfavored in bulk solution, thereby simultaneously lowering the radical oxidation potential and suppressing arene addition.^3^ This work uncovers a previously inaccessible, copper-free manifold for nucleophilic substitution at sterically congested carbon centers and expands how enzymes can catalyze C(sp^3^)–N bond formation with control over both stereo- and chemoselectivity.

Nature’s enzymes do not merely accelerate chemical reactions; they sculpt precise constellations of non-covalent interactions that stabilize one transition state among many.^4^ These interactions are so precise that even subtle perturbations can completely alter the energy landscape and divert the reaction onto a different mechanistic pathway.^5^ For instance, mutation of a conserved glutamate to tyrosine in a retaining β-glycosyltransferase (GTs) abolishes the canonical double-S_N_2 mechanism, remodeling the active site to enable front-face S_N_i-like reactivity via oxocarbenium ion stabilization (Fig. 1A).^5,6^ Such cases illustrate how enzymes can channel intermediates into distinct reactivity. However, evolving and reprogramming enzymes to reliably switch chemoselectivity between distinct, competing functional groups while simultaneously controlling enantioselectivity remains a formidable challenge.^7,8^

α-Tertiary amines are an increasingly important motif in bioactive molecules because of their metabolic stability and hydrogen bonding ability.^9,10^ Yet stereoselective synthesis of these structures via intermolecular C(sp^3^)–N bond construction remains challenging. Classical bimolecular substitution pathways are ineffective with tertiary electrophiles owing to prohibitive S_N_2 barriers and competing elimination.^11,12^ Copper-catalyzed systems have addressed this challenge through single-electron pathways in which alkyl halides are reduced to radicals that combine with Cu–anilido intermediates to forge C(sp^3^)–N bonds (Fig. 1B).^2,13–16^ Although powerful, these copper(I)-anilidyl radical intermediates have significant radical character on both the nitrogen and aryl ring of the aniline motif,^2^ and consequently require *para*-substituents to avoid arene functionalization (Fig. 1B).

Biocatalysts are widely used to prepare chiral compounds because of the wealth of enzymes capable of catalyzing formal reductive amination reactions with high levels of enantioselectivity.^17–19^ However, these catalysts are mechanistically limited to α-secondary amine formation and cannot directly access α-tertiary centers.^20^ Increasingly, chemists are seeking to transcend such constraints by introducing non-natural catalytic logic into proteins, including *N*-centered radicals,^17^ metal-nitrenoid intermediates,^21–23^ and metal-anilidos.^24^ Notably, these approaches largely recapitulate mechanisms established in small-molecule catalysis and have similar synthetic limitations. In contrast, evolvable non-covalent interaction networks within protein active sites offer the opportunity to unlock fundamentally new reaction mechanisms. Such emergent pathways could circumvent the intrinsic limitations of existing C–N bond-forming strategies and expand how we use enzymes in chemical synthesis.^25^

In this context, we recently demonstrated that engineered Baeyer–Villiger monooxygenases catalyze an asymmetric intramolecular hydroamination.^3^ Mechanistically, C–N bond formation proceeds through an enzyme-templated interaction between a benzylic radical and the pendant aniline. This organized radical–arene association increases the radical’s effective reducing power, enabling oxidation by the flavin cofactor and concomitant C–N bond formation. However, this mechanism was only available to cyclization reactions, where limited conformational freedom minimizes the requirement for precise control of intermolecular orientation. We therefore questioned whether a protein active site could enforce analogous radical–amine interactions in an intermolecular coupling reaction, thereby controlling chemo- and enantioselectivity without reliance on metal–ligand coordination.

As an initial model system, we explored the coupling of unsubstituted aniline (**1**) with tertiary α-bromoamide **2** to afford an *α*-tertiary amino amide (**3**) (Fig. 2A). This system is attractive because nucleophilic substitution reactions cannot occur on the alkyl halide. Unsubstituted anilines represent a problematic substrate for metal-based coupling methods. Indeed, under traditional photoredox conditions (with tris(2,2’-bipyridine)ruthenium(II) as catalyst), the C-alkylated product (**4**) is formed with 80% chemoselectivity and only 2% yield of the *N*-alkylated product (Fig. 2B). Notably, other photoredox catalysts failed to afford the product in detectable yields (Fig. S3). Next, we tested oxidoreductase families that were previously shown to have untapped photoenzymatic functions.^26^ Using ketoreductases under cyan-light irradiation (490 nm) led to the formation of the undesired C(sp^3^)–C(sp^2^) arylation adduct (**4)** with 80% chemoselectivity (Fig. 2B and Fig S4). However, flavin-dependent ‘ene’-reductases (EREDs) provided a mixture of *C*- and *N*-alkylation (**3,** Fig S2), with morphinone reductase (MorB) producing the desired product in 28% yield as a 66:34 ratio of *N-*alkylation vs *C*-alkylation **3:4** with the *N*-alkylated product formed in 52:48 e.r (Fig. S5). Control experiments confirmed that omitting visible-light irradiation under otherwise optimal conditions resulted in no detectable product formation (Fig. 2C). Exclusion of the enzyme likewise abolished conversion entirely, ruling out non-enzymatic background pathways. Finally, the free flavin cofactor failed to produce either *N*-alkylation product (**3**) or *C*-alkylation (**4**) under photoenzymatic conditions, indicating that the protein active site was required for this reactivity.

Next, we optimize the protein using iterative site-saturation mutagenesis to enhance the catalytic performance of MorB by improving the yield, chemoselectivity, and enantioselectivity.^7,27^ Initial efforts focused on active-site residues proximal to the flavin cofactor and substrate-binding pocket; for the first round, we selected 12 amino acid residues. Substitution of an NADPH-binding residue N189 to histidine markedly enhanced yield—a logical outcome given our pursuit of non-native SET reactivity rather than the enzyme’s canonical hydride transfer mechanism.^28^ This single substitution delivered the product in 42% yield, 75:25 *N:C*-alkylation, and 53:47 e.r. (Fig. 2D).

Subsequent rounds identified V73N, which increased yield (58%) and chemoselectivity (80:20 *N:C*-alkylation) for the model reaction; F246Y was found to be crucial for increased enantioselectivity (72:28 e.r.) while retaining reactivity and chemoselectivity. Further mutagenesis with S353T improved reactivity, reaching 72% yield and 83:17 *N:C*-alkylation, while I59V delivered 78% yield, 83:17 *N:C*-alkylation chemoselectivity, and 81:21 e.r. A final A134V substitution provided the optimal variant, affording the N-alkylated product in 76% yield, 87:13 *N:C*-alkylation chemoselectivity, and 88:12 e.r. (Fig. 2D). The final mutant scaled robustly to 0.1 mmol, affording 48% yield while retaining high chemoselectivity (84:16 *N:C-*alkylation), and enantioselectivity (86:14 er.) (Fig. S10). The catalytic efficiency was further evaluated using cell-free lysate, which produced the desired product in 25% yield (82:18 *N:C-*alkylation, 86:14 er.) with a reduced enzyme loading of 0.2 mol% (Table. S3).

With the evolved enzyme and optimized reaction conditions in hand, we sought to explore the substrate scope across diverse anilines and tertiary alkyl halides. For the tertiary α-bromoamide derivatives (**2**), α-benzyl substituents spanned electron-donating and electron-withdrawing substituents at the *meta* and *para* positions, in yields ranging from 18–78%, high chemoselectivity favoring the *N*-alkylation (>70:30 *N:C*-alkylation), while maintaining good enantioselectivity (60:40–84:16 er.). While afforded viable reactivity, the ortho-substituents resulted in eroded chemoselectivity values; *ortho*-methyl substitution yields 66:34 *N:C*-alkylation, attributable to steric disruption of key π-stacking interactions with the aniline (see mechanistic studies below). This enzyme, however, was limited to α-methyl substitution; homologation to an α-ethyl group completely suppressed reactivity (Fig. S10, 11), although directed evolution could potentially address this limitation. Notably, this mutant can also accommodate tertiary α-bromomethyl ester (**29)**, furnishing the coupled product in 30% yield, 72:28 *N:C*-alkylation chemoselectivity and 72:28 e.r.

The aniline scope spanned electron-rich and electron-deficient derivatives, with *para*-substituents consistently outperforming their *meta*- and *ortho*- counterparts (up to 78% yield, 89:11 er, and N-alkylation exclusively). Notably, *para*-substituted anilines proceeded with no detected arylation byproducts; this regiochemical specificity prompted the hypothesis that arene addition, when operative, occurs selectively at the *para* position. To test this, we synthesized the para-alkylated arylation product **4** via chemical routes and confirmed its formation in the reaction by co-injection with reaction mixtures (supplementary Section 2.5), validating the site of competing arene addition. *Meta*-substituted anilines provided good yields (24–58%) with synthetically useful e.r. (up to 90:10 er). The chemoselectivity of meta-substituted anilines varied significantly, with good selectivity for electron-donating substituents, while selectivity dropped for electron-deficient anilines (see mechanistic discussion section below). Ortho-substituted anilines showed further erosion in chemoselectivity, consistent with steric occlusion of the nitrogen lone pair and additional steric and electronic perturbations that are dissected in the mechanistic analysis section. N-Methylaniline proved similarly compatible, affording the tertiary amine in 52% yield with 92:8 e.r. and 72:28 *N:C*-alkylation. This active-site tolerance for N-substitution broadens access to *N, N*-dialkylated anilines prevalent in medicinal chemistry.^29^

We observed that aniline arene substituents exerted a substantial effect on chemoselectivity, spanning a wide range from (*m*-CN, 50:50 *N:C*-alkylation) to (*m*-*t*Bu, 93:7 *N:C*-alkylation). Preliminary examination shows that both electron-donating and sterically bulky groups favored the C(sp^3^)–N product, revealing substantial diversity in reactivity across simple aniline derivatives (Fig. 3). To dissect electronic contributions, we analyzed *meta*-substituted anilines using Hammett σ_m_ parameters, observing a weak negative correlation (R^2^ = 0.64) suggestive of partial positive charge buildup in the selectivity-determining transition state (Fig. S30).^30–32^ This modest correlation suggests that additional factors dictate selectivity at the transition-state determining the selectivity.

Consequently, we employed multivariate statistical modeling to elucidate further the interplay between electronic and steric factors governing chemoselectivity. Stepwise linear regression statistical modeling of the aniline library yielded a robust, highly predictive two-parameter model (R^2^ = 0.88, Fig. 4A). This model correlates *N*-selectivity with the natural bond orbital (NBO) charge on the aniline nitrogen and the Sterimol B_1_ minimum width of the aniline ring (Fig. 4B).^33,34^ Higher chemoselectivity arises from orthogonal steric and electronic effects: elevated B_1_ values signify increased steric bulk that shields the arene π-face, impeding undesired C(sp^3^)–C(sp^2^) addition, while more negative NBO charges reflect enhanced aniline nucleophilicity that accelerates C–N bond formation. This quantitative structure–selectivity relationship rationalizes the enzyme’s bias toward *N*-alkylation. However, the electronic NBO effect is more nuanced: increased aniline nucleophilicity (more negative nitrogen charge) simultaneously (1) reduces π-system’s propensity for radical addition,^35^ and (2) stabilizes key transition-state interactions— including radical hyperconjugation and π-stacking with the substrate (Fig. 4C).^3^ This multifaceted electronic control reveals how subtle nucleophilicity gradients can dictate reaction outcomes in radical C–N coupling.

When considering the mechanism of C–N bond formation, we considered three possibilities: (1) a radical-radical coupling mechanism where the alkyl radical reacts with aniline radical cation, (2) oxidation of an intermediate where the aniline HOMO interacts with the alkyl radical by a flavin species, or (3) radical polar crossover. A radical polar crossover pathway is unlikely because we do not observe a hydroxylated product under any of the reaction conditions examined, despite water being present as a potential nucleophile (Fig. S22). We explored the ability of flavin to oxidize aniline by using it as an electron donor in the photoreduction of flavin quinone. Upon photoexcitation of the optimal variant in the presence and absence of aniline, we observed no difference in the rate of flavin reduction, suggesting that aniline is not oxidized by the flavin quinone (Fig. S14, 15). Instead, these data support a mechanism where either the flavin semiquinone or hydroquinone reduces the alkyl halide to generate a tertiary radical that forms a hyperconjugative interaction with aniline (referred to as the C–N radical intermediate), enabling oxidation by flavin to form a product with concomitant formation of reduced flavin. We envision this C–N bond-forming step as being the microscopic reverse of C–N mesolytic cleavage.

We perform DFT optimization of the product and the C–N radical intermediate at the M062X/6–311+G(d,p) level of theory to investigate the mechanism of C–N bond formation (Fig. S23).^36^ This analysis pinpointed the critical interactions stabilizing the transition state for the aniline addition to the radical, featuring charge buildup on nitrogen and a prominent π-stacking interaction between the aniline ring and the benzyl group of the substrate (Fig. 4D, Fig. S24). This observation was further confirmed using the computed noncovalent interaction (NCI) plot (Fig. 4D).^37^ Such noncovalent interactions are known to be substantially strengthened within the confined microenvironment of enzyme active sites, where enhanced binding energies amplify their stabilizing effect.^38,39^ We thus postulated that these interactions govern chemoselectivity.

These mechanistic insights rationalize the counterintuitive chemoselectivity difference between 3,5- and 2,3-dimethylanilines (26, 27). Despite similar electronics and greater C-_*para*_ steric shielding in the 3,5-isomer, experimental data reveal higher selectivity for the 2,3-substrate. We attribute this to intermolecular π-stacking in the 2,3-isomer that stabilizes the selectivity-determining transition state, whereas the 3,5-dimethyl groups disrupt these critical interactions (Fig. 5E). Similarly, homo-benzylic extension (phenethyl) or methylene abolishes optimal π-stacking geometry, dramatically eroding *N:C* ratios (Fig. 4E). *Ortho*-substituted halides further compromise selectivity by distorting benzyl planarity and disrupting orbital overlap with the aniline π-system. Collectively, these observations establish π-stacking as the selectivity linchpin, providing precise control over reaction outcomes that small-molecule catalysts cannot achieve.

Next, we conducted a series of experiments to determine the mechanism of this transformation. Our initial hypothesis was that flavin quinone is photochemically reduced to the flavin hydroquinone, which reduces the alkyl halide to produce a tertiary radical and flavin semiquinone. The acyl radical would then couple with the aniline nitrogen lone pair to form a delocalized radical intermediate, followed by single-electron oxidation by semiquinone to yield the product and regenerate hydroquinone. To test this hypothesis, we reduced the evolved enzyme to the hydroquinone oxidation state using sodium dithionite. When the reduced enzyme was supplied with substrates in the dark, no reaction was observed, indicating that ground-state flavin hydroquinone cannot initiate this reaction (Fig. 5B, Fig. S19). When irradiated with light, the reduced enzyme formed the product in low 14% yield and 58:42 *N:C*-alkylation.^40^ Replacing sodium dithionite with 30 mol % NADPH afforded the product in 29% yield and 69:31 *N:C-*alkylation (Fig. S21). As the selectivity is significantly lower than what is observed under the standard reaction conditions, we conclude that the hydroquinone is not responsible for radical initiation.

Our group previously found that flavin semiquinone can reduce alkyl halides.^41^ Based on this reactivity, we questioned whether initiation from the semiquinone could account for the observed reactivity. The final mutant was photoreduced to the flavin semiquinone under cyan light in tricine buffer (supplementary S7.5),^41^ after which substrates **1** and **2** were added in the dark. Under these conditions, no product was detected (Fig. 5B). As the hydroquinone is unreactive in either the ground or excited state and the semiquinone is unreactive in the ground state, we conclude that substrate reduction likely occurs from the flavin semiquinone excited state.

Taken together, we hypothesize that photoexcited flavin semiquinone initiates single-electron reduction of the tertiary alkyl halide, generating a tertiary radical that couples selectively with aniline via protein-templated non-covalent interactions (Fig. 5A). The formation of the α-acyl radical intermediate (IM1, Fig. 5A) was further substantiated by a radical trapping experiment with DMPO (5,5-dimethylpyrroline N-oxide). Performing the reaction under standard conditions in the presence of DMPO markedly reduced the yield of **3** (14%) and yielded a DMPO–adduct in a 1:1 ratio with the *N*-alkylation product (supplementary Section 6.3).

To elucidate the ensuing α-acyl radical–aniline coupling step, we drew inspiration from our recent intramolecular photoenzymatic alkene hydroamination. In that system, an enzyme-bound benzyl radical engaged a neighboring amine lone pair, delocalizing spin density and attenuating the radical oxidation potential to promote cyclization. By analogy, we propose an intermolecular mechanism in the present system involving hyperconjugation between the tertiary α-acyl radical and the aniline nitrogen lone pair. This interaction is proposed to (1) lower the radical’s oxidation potential, thereby favoring selective C–N bond formation and (2) generate a partial positive character on the nitrogen, allowing noncovalent stabilization.

Consistent with this model, substrate profiling revealed that aliphatic amines and phenols were unreactive, supporting specific noncovalent interactions and hyperconjugation as the primary determinant of reactivity and selectivity rather than a generic radical polar crossover process that would generate an α-carbocation reactive towards diverse nucleophiles.

We quantified the impact of π-stacking and hyperconjugation using density functional theory to identify and visualize frontier molecular orbitals stabilizing the α-acyl radical–aniline encounter complex (**IM2**, Fig. 5A). Born–Haber cycle calculations revealed a substantial decrease in the oxidation potential for the preorganized complex (ΔE_ox_ = −0.49V versus SCE) by comparison to the radical alone, suggesting that a noncovalent *n→SOMO* interaction with the aniline modulates the redox potential of the substrate (Fig. 5C).

The optimized adduct structure (**IM2**, Fig. 5A) at the M06–2X/6–311+G(d,p) level of theory revealed π-stacking alongside an intermolecular hydrogen bond between the aniline and the carbonyl (Fig. S26). This geometrically precise arrangement of the intermediates is entropically disfavored without enzymatic confinement.^42^ Enzyme active sites are known to accelerate reactions using hydrogen bonding networks involving amino acid side chains and water molecules.^43,44^ We modeled in explicit water molecules and found that the addition of two waters formed a defined encounter complex that decreased the N−radical distance from 3.8 Å to 3.1 Å (Fig. 5C). Water-mediated bridging further lowered the radical oxidation potential (ΔE_ox_ = −1.19 V versus SCE), improving orbital overlap that accelerates enzymatic C–N coupling by orders of magnitude relative to bulk solution.

Frontier molecular orbital analysis of the two-water-bridged complex revealed pronounced antibonding character between the α-acyl carbon SOMO and aniline nitrogen lone-pair orbitals (Fig. 5D). The HOMO has strong bonding between the radical-bearing carbon and aniline nitrogen, indicating *n→SOMO* hyperconjugative stabilization directs selective C_(sp3)_–N coupling. This orbital alignment, with the compressed 3.1 Å N–C separation, provides a clear electronic basis for the enzyme’s chemoselectivity—funneling the radical toward productive bond formation over arene addition.

Structure prediction and docking for the evolved variant with *ProteinX* suggest that two active-site histidines could serve as the bridging hydrogen bonding residues within the protein active site (Fig. 5E). The N189H mutation—identified in our evolution campaign—increased yield from 28% to 42% and selectivity from 66% to 75%, enabling His189 to pair with conserved His186 to work in tandem for substrate bridging. Site-saturation mutagenesis of His186 in the final evolution round sharply reduced reactivity and selectivity. These insights from the engineering campaign suggest the important role of the His189–His186 residues in templating selective C(sp^3^)–N coupling.

In conclusion, we demonstrate a photoenzymatic access to α-tertiary amines via active-site templating, enabling enanioconvergent C(sp^3^)–N reactivity without metal coordination. We envision this copper-free radical nucleophilic substitution mechanism diversifying radical aminations for challenging tertiary substrates.

## Supplementary Material

This is a list of supplementary files associated with this preprint. Click to download.

- model2026.csv
- RedoxCalculations.xlsx
- SupplementalInformation.pdf
- SIDFT.zip
- Codeformultivariatemodel.docx

## Figures and Tables

**Fig. 1. |:**
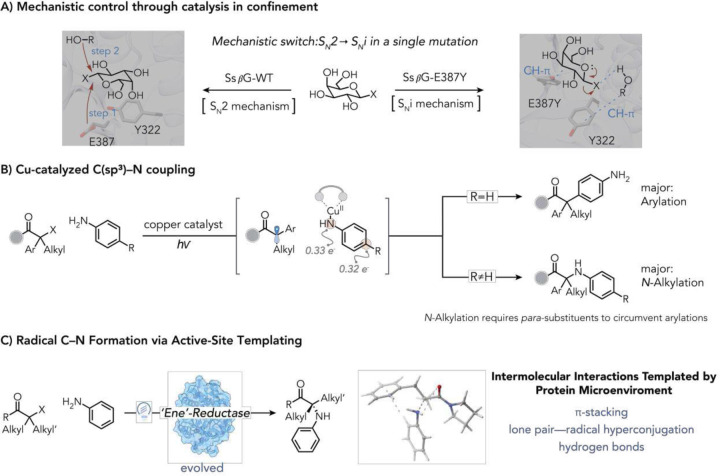
Mechanistic divergence unlocks α-tertiary amine synthesis. **(A**) active-site confinement enables mechanistic shifts in retaining β-glycosyltransferase. **(B**) Cu-catalyzed C(sp^3^)–N coupling limited by arene radical addition pathways. (**C**) Evolved flavin enzyme enables selective intermolecular radical–aniline coupling via non-covalent templating.

**Fig. 2. |:**
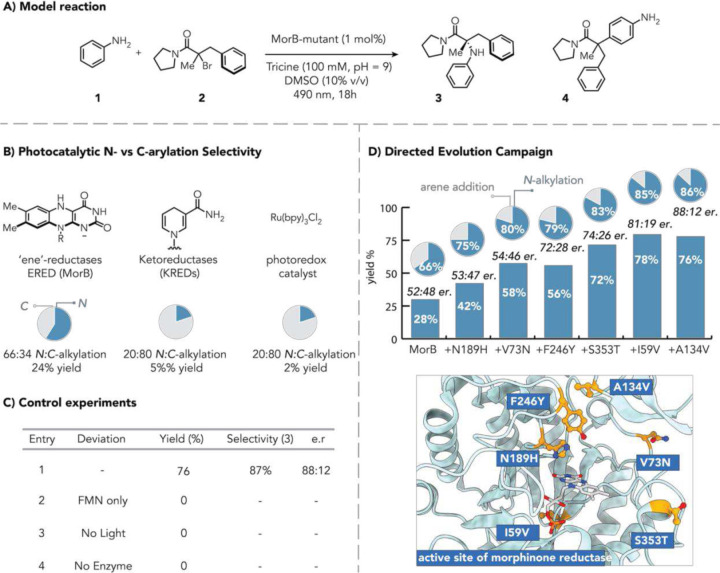
Directed evolution enables selective C(sp^3^)–N coupling. **(A)** Designing a model reaction. **(B)** Photocatalyst systems comparison (yields and selectivities are averaged from a library of catalysts, see supplementary Fig. S3, 4, 5). **(C)** Control experiments assessing the effect of reaction components on catalysis. **(D)** Evolution campaign summary and active-site mutations on MorB parent structure.

**Fig. 3. |:**
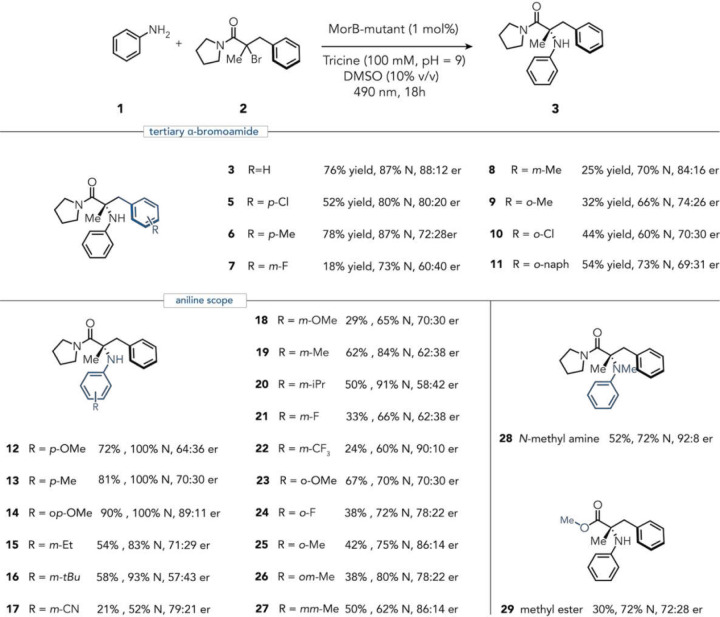
Reaction scope. Substrate modifications are organized into three sections: substituents on the tertiary bromide, aniline derivatives, and additional structural variations not on either aniline or the benzyl ring.

**Fig. 4. |:**
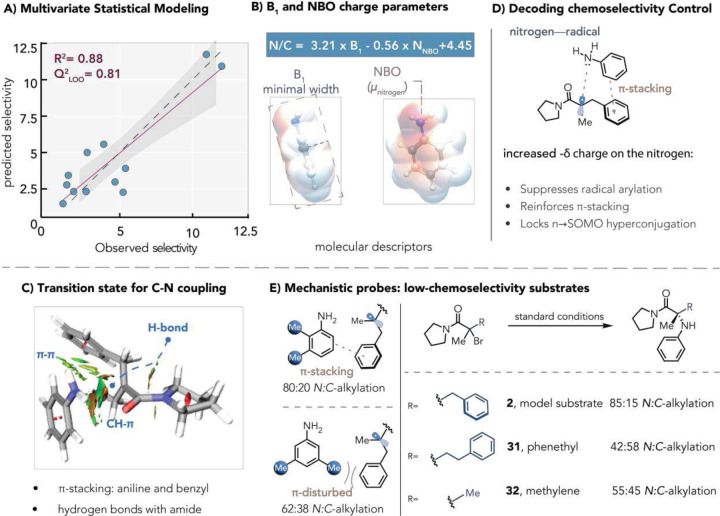
Structure–activity relationships and interactions governing chemoselectivity. **(A)** A multivariate linear regression model correlating chemoselectivity against B_1_ and NBO charge at the nitrogen, cross-validation using LOO (Leave-One-Out) was conducted. Normalized equation **(B**) Description of feature parameters dictating selectivity. **(C**) Analysis of the electronic effect on chemoselectivity with three hypotheses. **(D)** Noncovalent interaction (NCI) visualization of the C-N bonding transition state, where sheets represent attractive forces. **(E)** Analysis of poor and unreactive substrates.

**Fig. 5.**
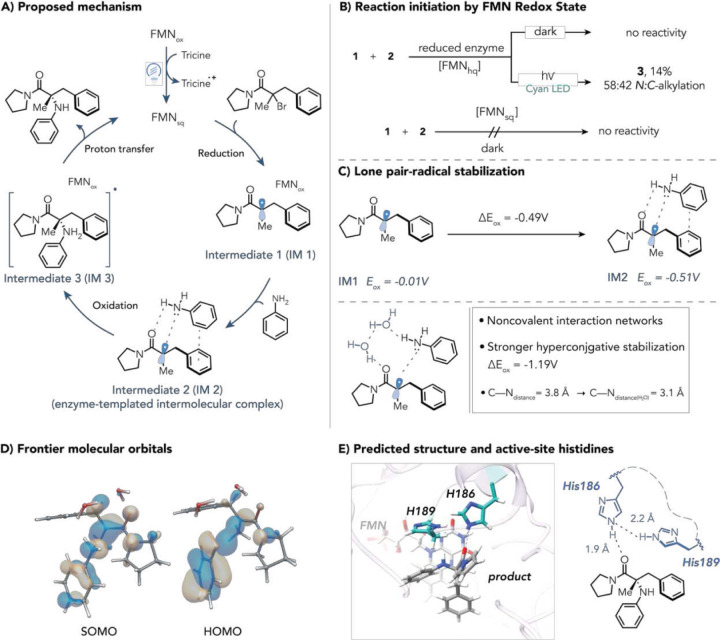
Mechanism evaluation. **A**) proposed catalytic cycle. **B**) mechanistic probes shedding light in redox state initiating the reaction. **C**) Redox potentials were determined using the Born–Haber relation on the DFT optimized structures at the M06–2X/6–311+G(d,p) level of theory. The water bridging mechanism further stabilizes the noncovalent interactions. **D**) Frontier molecular orbital visualization, HOMO (highest occupied molecular orbital), SOMO (singly occupied molecular orbital). **E**) final mutant structure determination and substrate docking using *ProteinX*, and an illustrative ChemDraw of the product in the active site.

## Data Availability

Data are available in the supplementary materials.
